# Were there losses in social support during the pandemic? Testing the impact of COVID-19 on psychological adjustment to trauma in United States adults

**DOI:** 10.3389/fpsyg.2022.1061621

**Published:** 2022-12-22

**Authors:** Benjamin J. Mitchell, Emily A. Gawlik, Brittany J. Baugher, Richard L. George, Farid F. Muakkassa, Ali F. Mallat, John Gunstad, Douglas L. Delahanty, Karin G. Coifman

**Affiliations:** ^1^Department of Psychological Science and Public Health, Kent State University, Kent, OH, United States; ^2^Summa Health Systems, Akron, OH, United States; ^3^Department of Surgery, Northeast Ohio Medical University, Rootstown Township, OH, United States; ^4^Cleveland Clinic Akron General, Akron, OH, United States

**Keywords:** social support, psychological adjustment, trauma, depression, post-traumatic stress, COVID-19

## Abstract

**Introduction:**

Social support is a key protective factor in the psychological adjustment of individuals to traumatic events. However, since March 2020, extant research has revealed evidence of increased loneliness, social isolation, and disconnection, likely due to COVID-19 pandemic-related recommendations that restricted day-to-day contact with others.

**Methods:**

In this investigation, we applied a case-control design to test the direct impacts of the pandemic on social support in United States adults recovering from a significant injury caused by PTSD-qualifying, traumatic events (e.g., motor vehicle crashes, violence, etc.). We compared individuals who experienced trauma during the pandemic, the “cases” recruited and evaluated between December 2020 to April 2022, to trauma-exposed “controls,” recruited and evaluated pre-pandemic, from August 2018 through March 9, 2020 (prior to changes in public health recommendations in the region). Cohorts were matched on key demographics (age, sex, education, race/ethnicity, income) and injury severity variables. We tested to see if there were differences in reported social support over the first 5 months of adjustment, considering variable operationalizations of social support from social network size to social constraints in disclosure. Next, we tested to see if the protective role of social support in psychological adjustment to trauma was moderated by cohort status to determine if the impacts of the pandemic extended to changes in the *process of adjustment*.

**Results:**

The results of our analyses suggested that there were no significant cohort differences, meaning that whether prior to or during the pandemic, individuals reported similar levels of social support that were generally protective, and similar levels of psychological symptoms. However, there was some evidence of moderation by cohort status when examining the process of adjustment. Specifically, when examining symptoms of post-traumatic stress over time, individuals adjusting to traumatic events during COVID-19 received less benefit from social support.

**Discussion:**

Although negative mental health implications of the pandemic are increasingly evident, it has not been clear how the pandemic impacted normative psychological adjustment processes. These results are one of the first direct tests of the impact of COVID-19 on longitudinal adjustment to trauma and suggest some minimal impacts.

## Introduction

The COVID-19 pandemic has indisputably had significant consequences for physical and mental health. In addition to the threat posed by the virus itself, individuals have experienced disruptions to daily living on a massive scale, including changes in work or employment status, economic difficulties, and other financial hardships (Panchal et al., 2021). That is notwithstanding the highly significant loss of life and long-term medical disability associated with contracting the disease itself (Hodgson et al., 2021; Lopez-Leon et al., 2021). Moreover, these pressures have created even greater difficulties for individuals already contending with other stressors (Manchia et al., 2022). Large-scale community disasters like pandemics have been shown to threaten otherwise adaptive adjustment processes (Goldmann and Galea, 2014). In particular, diminished access to social resources that might otherwise help to mitigate stress could greatly compound the risk for mental illness (Belsher et al., 2012). In this investigation, we sought to explicate the impacts of the COVID-19 pandemic on social support processes in adults recovering from injuries sustained during traumatic events (e.g., motor vehicle crashes, violence, industrial accidents, etc.).

Social support is a consistent and highly protective factor in psychological adjustment to traumatic events as demonstrated by decades of research (Cohen and Wills, 1985; Joseph, 1999; Guay et al., 2006). We focused specifically on social support, given explicit, broadly administered social recommendations that served as countermeasures to slow the spread of the COVID-19 virus, including stay-at-home orders, self-isolation/quarantine periods, limited public social interactions, and being otherwise less likely to see close friends and other important social contacts (Centers for Disease Control, 2020). These “social distancing” recommendations may have impacted individuals’ experiences of social support (Mancini, 2020). As prevailing theories suggest that social support serves a “buffering” effect on adjustment to stress, understanding to what degree the pandemic has impacted its influence on psychological adaptation to traumatic events is critical (Cohen and Wills, 1985; Arnberg et al., 2012; Evans et al., 2013).

### Social support and recovery from trauma

Social support, often operationalized as the perceived receipt of both emotional and instrumental aid from close others, has been consistently associated with psychological adaptation to traumatic events by facilitating resilience and recovery in a variety of ways (Schwarzer and Knoll, 2007; Cohen and Wills, 1985). Prevailing theories of social support suggest that it has a “buffering” effect on stress, reducing reports of negative affect and psychological distress (Viswesvaran et al., 1999; Werner-Seidler et al., 2017). Indeed, this has been shown even in individuals at particularly high risk for mental illness due to frequent trauma exposure (e.g., first responders: Prati and Pietrantoni, 2010). Moreover, increasingly, research suggests that social support benefits may operate *via* behavioral *and* biological channels, including reducing the decremental toll of stress on key systems (e.g., neuroendocrine; inflammatory pathways, c.f. Uchino, 2006). In short, social support has been shown to be a reliable protective factor for individuals during adjustment to highly stressful life circumstances, reducing distress, enhancing well-being, and facilitating physical and mental health.

Past research has operationalized social support in a variety of ways. For example, there are differences in the relative benefits of *functional* social support, an individual’s subjective experience of support, versus *structural* social support, a quantified index of one’s social network (Stewart et al., 2022). Further, the extent to which individuals feel unconstrained in seeking support from others may also contribute to psychological adaptation through different pathways. For example, considerable prior research has suggested that the degree to which an individual feels they can disclose difficulties to a partner or supportive other can influence adaptation to a significant stressor (e.g., cancer Lepore and Revenson, 2007; Chu et al., 2021). Hence, *social constraints against disclosure* are another unique pathway by which social support can be considered.

### COVID-19 and social support

There is emerging evidence that social support has been associated with more favorable outcomes during the COVID-19 pandemic. Across a variety of samples and circumstances, perceived social support in particular, has been found to be protective against high levels of loneliness and psychological symptoms including depression and post-traumatic stress (e.g., Bu et al., 2020; Gloster et al., 2020; Grey et al., 2020; Zaken et al., 2022). For example, a multi-wave longitudinal study of Chinese adults demonstrated that higher perceived social support was protective against increased anxiety in individuals high in loneliness and moderated the relationship between loneliness and anxiety during the pandemic (Xu et al., 2020). Although social support has been protective during the pandemic, there is also evidence that social support was adversely affected by the COVID-19 pandemic. For example, increased reports of isolation and reduced contact have been associated with increases in depression (e.g., Kotwal et al., 2021; Pancani et al., 2021); increased substance use (e.g., Bartel et al., 2020) and declines in cognitive function (e.g., Ingram et al., 2021). However, despite reports of loneliness and declining social support in the general population, there is also evidence of considerable individual differences in adjustment to the circumstances of the pandemic (Mancini, 2020) and only mixed evidence of broader psychological impacts (Prati and Mancini, 2021).

## The current investigation

The current study sought to test the contextual impact of the COVID-19 pandemic on social support processes and psychological adjustment. Because the protective role of social support may be most evident in the aftermath of acute stress (Orlas et al., 2021), we focused our investigation on adults admitted to the hospital following injury caused by traumatic events (e.g., serious motor-vehicle crashes, violence, etc). We applied a case–control design defined by context so that comparisons could be made between a “control” cohort recruited *before* the active phase of the pandemic in the United States (2018 to March 9, 2020) and a “case” cohort recruited *during* the pandemic (December 2020 to April 2022). Specifically, our twofold goal was to examine whether (1) experiences of social support were affected by the pandemic and (2) whether the *process of adjustment* was influenced by any resulting changes in social support. Keeping with a nuanced conceptualization of social support, we considered three different operationalizations: perceived social support, social network size, and report of social constraints. Given prior evidence suggesting the unique impacts of the pandemic on particular groups (e.g., lower SES groups, the elderly; Patel et al., 2020; Grolli et al., 2021), we matched our samples carefully based on demographic, economic, and injury-related characteristics to ensure a rigorous comparison. Moreover, we not only tested cohort differences in social support and psychological health indicators over time, but also tested potential moderation by COVID-19 context in the expected associations between social support and psychological adjustment over a 5-month period. For these latter tests, we expected, based on extensive prior research, that early reports of social support (perceived social support, social network size) would predict fewer psychological symptoms at 5-months post-trauma, but that social constraints on disclosure would predict higher symptoms. Although we did not develop any specific *a priori* predictions for whether COVID-19 would impact adjustment or not, we did anticipate that any impact would likely be deleterious to adjustment.

## Materials and methods

### Participants

United States adults were recruited for this investigation based on recent admission to the hospital following injury caused by traumatic events (e.g., 45.2% serious motor vehicle crashes, 10.7% violence, 39.7% other severe accidents). Eligibility was determined based on the type of event (conforming with current DSM-5 criteria for PTSD diagnosis, American Psychiatric Association, 2013), age between 21 and 65, English fluency, and the absence of current treatment for substance dependence or psychosis. Participants were recruited at inpatient and outpatient clinics at one of two American College of Surgeons verified Level 1 trauma hospital centers located in an urban area in the Midwest, United States. within 1–2 weeks of the traumatic event. Patients were invited to participate in a study “investigating how people adjust following a significant injury.” Participants in each of the two cohorts for this investigation were selected from the larger sample of the parent-project based on dates of recruitment that conformed to the timing of the COVID-19 pandemic. The pre-COVID-19 cohort was recruited from August 2018 through early 2020, and all relevant pre-COVID-19 data collection occurred prior to March 9, 2020, approximately 4 days before public health recommendations and related COVID-19 shut-downs were enforced nationally and in the local region (Cucinotta and Vanelli, 2020). The COVID-19 cohort included participants recruited from December 2020 through April 2022, corresponding to several peaks in disease incidence in the region.

From the parent sample, *n* = 81 participants had completed study procedures prior to the onset of the COVID-19 pandemic and *n* = 42 completed the study procedures during the COVID-19 pandemic. All *n* = 42 participants in the COVID-19 cohort were selected as the “cases” (COVID-19 cohort), and *n* = 42 participants of the *n* = 81 members of the pre-COVID-19 cohort were selected as “controls,” based on matched age, gender, education, reported income, race/ethnicity, and injury severity status [assessed using injury severity scores (ISS)]. Independent-samples *t*-tests and chi-square tests were conducted to confirm the matching criteria and showed no differences in the two study cohorts (see Table 1). Across both cohorts (*N* = 84), the mean age was 41.54 (*SD* = 1.36), with 48.8% (*n* = 41) of participants self-identifying as female and 51.2% identifying as male (*n* = 43). Most participants self-identified as White (*n* = 72; 85.7%), with 12 identifying as Black or African American (14.3%), 5 identifying as American Indian or Alaska Native (6%), and 1 identifying as Asian (1.2%). In addition, 3 participants (3.6%) self-identified as Hispanic, Latino, or of Spanish Origin. In total, 7 participants (8.3%) completed some high school, 37 participants (44%) finished high school (or GED) and/or completed some college, 16 participants (19%) completed an associate’s degree, 9 participants (10.7%) completed a bachelor’s degree, 10 participants (11.9%) completed a graduate degree, and 3 participants (3.6%) responded with “other” (e.g., vocational school). Of the 77 participants who reported annual family income, 24 participants (28.6%) reported earning less than $35,000, 8 participants (9.5%) reported earning between $35,000 and $49,999, 15 participants (17.9%) reported earning $50,000 to $74,999, 9 participants (10.7%) reported earning $75,000 to $99,999, and 21 participants (25%) reported earning $100,000 or more.

**Table 1:** Sample characteristics and cohort comparisons.

	Pre-COVID-19	COVID-19	Statistic	*p*-value
Age	*M* = 42.83	*M* = 40.24	*t* = 0.95	*p* = 0.344
	*SD* = 12.19	*SD* = 12.77		
Injury severity	*M* = 7.98	*M* = 9.86	*t* = 1.29	*p* = 0.201
	*SD* = 6.51	*SD* = 6.40		
Gender			*χ^2^* = 1.19	*p* = 0.275
*Male*	*n* = 24 (57.14%)	*n* = 19 (45.24%)		
*Female*	*n* = 18 (42.86%)	*n* = 23 (54.76%)		
Years of education			*χ^2^* = 2.63	*p* = 0.854
*Some high school*	*n* = 5 (11.90%)	*n* = 2 (4.76%)		
*Finished high school/GED*	*n* = 9 (21.43%)	*n* = 7 (16.67%)		
*High school and some college*	*n* = 10 (23.81%)	*n* = 11 (26.19%)		
*Associate’s degree*	*n* = 7 (16.67%)	*n* = 9 (21.43%)		
*Bachelor’s degree*	*n* = 4 (9.52%)	*n* = 5 (11.90%)		
*Graduate degree*	*n* = 6 (14.29%)	*n* = 4 (9.52%)		
*Other (*e.g., *vocational)*	*n* = 1 (2.38%)	*n* = 2 (4.76%)		
Race			*χ^2^* = 0.94	*p* = 0.332
*White*	*n* = 35 (83.33%)	*n* = 38 (90.48%)		
*Non-white*	*n* = 7 (16.67%)	*n* = 4 (9.52%)		
Ethnicity			*χ^2^* = 0.37	*p* = 0.542
*Hispanic*, *Latino*, *or Spanish Origin*	*n* = 2 (4.76%)	*n* = 1 (23.81%)		
Income			*χ^2^* = 3.08	*p* = 0.544
*< $35*,*000*	*n* = 12 (28.57%)	*n* = 12 (28.57%)		
*$35*,*000 – $49*,*000*	*n* = 4 (9.52%)	*n* = 4 (9.52%)		
*$50*,*000 – $74*,*999*	*n* = 10 (23.81%)	*n* = 5 (11.90%)		
*$75*,*000 – $99*,*000*	*n* = 3 (7.14%)	*n* = 6 (14.29%)		
*$100*,*000 or more*	*n* = 9 (21.43%)	*n* = 12 (28.57%)		

*t*, *t*-statistic; χ^2^, chi-squared statistic.

Finally, the cohorts did vary in the types of events (e.g., motor vehicle crashes versus interpersonal violence) that occurred, and this may be due to changes in behaviors due to the pandemic (Sherman et al., 2021). For example, there were reports of fewer accidental blunt trauma injuries presenting to Level 1 Trauma clinics during the height of the pandemic (Sherman et al., 2021). These cohort differences in injury type were examined and reported in detail in the Supplementary Material and did not impact the results. Moreover, the mean injury severity based on ISS was 8.87 (SD = 6.49), ranging from 1 to 29 and did not differ between the cohorts.

### Procedure

Following recruitment in-hospital, participants provided written informed consent approximately 1 month after their injury and were asked to report key demographic variables and completed measures of social support indices and depression and post-traumatic stress symptoms. At 4 to 5 months post-injury, participants again reported on symptoms and perceived social support. At this time point, participants also engaged in a variety of cognitive-emotional tasks and interviews, but these were not considered in this investigation. Injury severity scores were abstracted from the electronic medical record, scored according to the Abbreviated Injury Scale (Association for the Advancement of Automotive Medicine, 2016) by certified trauma registrars. Participants were compensated up to $135 for their participation in these activities. All procedures were approved by the institutional review board governing human subjects research at Kent State University and adhered to all international statutes governing the ethical treatment of humans in research.

### Measures

#### Social support

Given prior research on social support and dominant conceptualizations that emphasize both instrumental and structural support, we assessed social support in three key ways.

***Perceived social support*** was assessed at 1 and 5 months using the Medical Outcomes Study Social Support Survey (Sherbourne and Stewart, 1991), which assesses the degree to which individuals perceive others to be available to assist them both functionally and emotionally and was developed for medical patient populations. Internal consistency was excellent at both time points (Time 1: *α* = 0.97; Time 2: *α* = 0.98), and mean social support reported across both cohorts (Time 1: *M* = 3.89, *SD* = 0.97; Time 2: *M* = 3.72, *SD* = 1.11), was consistent with other patient cohorts (Sherbourne and Stewart, 1991; Moser et al., 2012). Ratings ranged from 1.05 to 5.00 (time 1) and from 1.00 to 5.00 (time 2), with higher ratings indicating higher perceived social support.

***Social network size*** was assessed at 1 month using the Social Network Index (Cohen et al., 1997). Participants were asked to rate their interactions with close others in multiple domains (parents, siblings, close friends, co-workers, etc.) based on the frequency of interaction per week. This measure did not explicitly index in-person versus virtual (e.g., phone call) social interaction, but rather whether any interaction occurred. Answers were coded so that if there was at least 1 contact every 2 weeks in a particular domain (e.g., church or religious cohort) then the domain was assigned a score of 1. If contact was less frequent, it was assigned a 0. The total score was the sum of all possible domains (up to 9 possible), with larger scores indicating larger social networks. The mean for this sample was *M* = 4.73, *SD* = 1.44 (range 1–8), which was consistent with other samples (e.g., Rutledge et al., 2008; Almahmoud et al., 2016).

***Social constraints*** were indexed using the Social Constraints on Disclosure Scale (Lepore and Revenson, 2007) at 1 month post-injury. Social constraints were assessed in all nine social domains (same as those measured for social network), by asking participants to rate 5 questions designed to index how much they perceived close others within that domain to be available to them for emotional disclosure and support relating to their injury and difficulties. Ratings across domains were then averaged to get a reliable estimate of their overall experience with social constraints, where higher scores suggest higher social constraints. Internal consistency was calculated for each domain and ranged from adequate to good [*α* = 0.67–0.82; mean *α* = 0.77 (*SD* = 0.05)] and the overall mean, *M* = 2.09, *SD* = 0.66 (range 1–4), was consistent with other populations (Pasipanodya et al., 2012).

***Psychological symptoms*** were assessed at both the 1-and 5-month time points. Participants reported depression symptoms using the Center for Epidemiological Studies – Depression Scale (Radloff, 1977) and symptoms of post-traumatic stress using the PTSD Checklist for the DSM-5 (PCL-5: Weathers et al., 2013). Both instruments had excellent internal consistency (CES-D: α = 0.91–0.92; PCL-5: α = 0.93 at both time points). The mean CES-D score for the full sample at time 1 (*M* = 18.00, *SD* = 12.26) was higher than the established clinical cutoff score of 16 for the measure (Lewinsohn et al., 1997), suggesting the sample on average had clinically significant levels of depression symptoms 1 month after their traumatic injury. The mean CES-D rating dropped to the clinical threshold at 4–5 months after their injury date (*M* = 15.45, *SD* = 11.31). The mean PCL-5 scores (Time 1: *M* = 21.38, *SD* = 17.19; Time 2: *M* = 18.02, *SD* = 15.86) were below the established clinical cutoff score of 31 for PTSD (Blevins et al., 2015).

***Injury severity scores (ISS)*** were collected from the Trauma Registry (CDM™ TraumaBase V9© [Clinical Data Management, Inc. by ESO; Conifer, CO)] which compiles ISS in the standard fashion (Baker et al., 1974), using the Abbreviated Injury Scale (Association for the Advancement of Automotive Medicine, 2016). ISS is a widely used tool to index the extent of injuries to the physical body and is computed by taking the sum of the squares of the single highest Abbreviated Injury Scale score from up to 3 of 6 defined body regions; the lowest ISS for an injured patient is 1 and the highest is 75. All trauma registrars are formally trained to score injuries using this method. Mean ISS across both cohorts was *M* = 8.87, *SD* = 6.49 (range 1–29) corresponding to a level of severity consistent with the dominant mechanism of injury (i.e., motor vehicle crashes: Palmer, 2007). However, ISS was unavailable for *n* = 6 participants (five of whom were from the COVID-19 cohort) because of refusal to provide access to HIPAA info and lags in ISS scoring at the time of submission.

## Data analytic plan

First, as preliminary analyses, we confirmed the matching of the two cohorts, on demographic and injury severity variables (see Table 1). Next, we ran zero-order correlations between all key variables, followed by independent-samples *t*-tests and repeated measures analysis of covariance (ANCOVA) to determine whether the cohorts (pre-COVID-19 vs. COVID-19) differed on any of the key variables, including perceived social support at times 1 and 2, social network (time 1 only), social constraints (time 1 only), depression symptoms at times 1 and 2, and post-traumatic stress symptoms (PTS) at times 1 and 2. In addition, because of our modest sample sizes, we confirmed any null findings with tests of equivalence using the two one-sided tests (TOST) approach with the “TOSTER” package in R (Lakens et al., 2018). Equivalence tests allowed us to determine whether non-significant effects reflect true equivalence or, rather, were best explained as the result of underpowered analyses. This approach consisted of setting equivalence bounds that allowed us to test whether we could statistically reject the presence of meaningful differences (Lakens, 2017). In the absence of precedent for effect sizes in this context, equivalence bounds were set using the smallest effect size detectable by our sample with 80% power (Lakens, 2017). An *a priori* power analysis to determine the equivalence testing boundary was executed using G*Power 3.1 (Faul et al., 2007). This simulation indicated that we would have 80% power to detect an effect size of *d* = 0.55, hence equivalence bounds were set to *d* = −0.55 and *d* = 0.55. This boundary is largely consistent with clinical efficacy effect size recommendations (e.g., Jakobsen et al., 2014) and therefore is a useful estimate of clinically meaningful difference. We did consider matching variables as potential covariates if they correlated with any of the outcome indices, however, only age did and thus all other matching variables were not considered in analyses.

Next, we conducted longitudinal models to test whether the protective effects of social support on adjustment depended on the cohort (Pre-COVID-19 versus COVID-19). We conducted linear ordinary least squares (OLS) regressions with depression or PTS symptoms (time 2) as the outcome variable. Social support (time 1) was conceptualized as the primary independent variable and cohort (cases v. controls) as the moderator. For each model, we co-varied age and symptoms at time 1, which were added in step 1. The social support variable was added in step 2, cohort in step 3, and their interaction term in step 4. Variables were centered as recommended. We tested all three measures of social support at time 1 (perceived social support, social network, and social constraints) as predictors in separate models. Finally, we conducted linear OLS regression with perceived social support at time 2 as the main predictor of symptoms at time 2, while controlling for symptoms at time 1. For this model, we followed the same iterative approach as above and tested for moderation by cohort. For any significant moderation effects in the above models, we conducted tests of simple slopes and plotted the moderation by cohort.

Given the number of OLS regression models conducted, we applied the Benjamini-Hochberg False Discovery Rate (FDR) Correction to correct for Type 1 error (Benjamini and Hochberg, 1995). This method consists of rank-ordering value of ps of each independent variable within a set of statistical tests in ascending order (smallest to largest value of p), then applying the following formula to each variable: (*i*/*m*)*q, where *i* is the variable’s value of p rank, *m* is the number of variables within the set of hypothesis tests, and *q* is the critical value of p chosen to interpret statistical significance (or the FDR). The largest value of p within the set of variables that was lower than its corresponding Benjamini-Hochberg critical value (based on (*i*/*m*)*q) was then interpreted as the critical value of p for determining statistical significance. We applied this method for two sets of hypothesis tests: (1) the four OLS regression models with depression symptoms at time 2 as the outcome variable, and (2) the four OLS regression models with PTS symptoms at time 2 as the outcome variable. We chose an initial FDR of *p = 0*.05 (the value used for *q* in the above formula), and both sets of hypothesis tests contained 20 variables each (*m* = 20). The results of this approach indicated that (1) any tests with depression symptoms as the outcome with a value of p of *p* ≤ 0.003 should be interpreted as statistically significant, and (2) any tests with PTS symptoms as the outcome with a value of p of *p* ≤ 0.008 should be interpreted as statistically significant. See Supplementary Tables S1, S2 for a full breakdown of the Benjamini-Hochberg Method applied to these two sets of hypothesis tests.

## Results

### Preliminary analyses

As described in Table 1, each cohort was matched correctly on age, gender, race, ethnicity, income, and education. In addition, there was no difference in Injury Severity Scores. Note, there were differences in the type of traumatic events by cohort as described above. These are described in detail in the Supplementary Material and did not impact any analyses.

Results from the zero-order correlations (see Table 2) were largely consistent with expectations. Most social support variables were associated with depression and PTS symptoms at both time points. Perceived social support was negatively correlated with depression and PTS symptoms at both time points. Lower social constraints (SC) was associated with lower depression and PTS symptoms at both time points. Social network was inversely correlated with depression at time 1. Injury severity was not associated with any variable, but age was inversely associated with perceived social support at both time points. Thus, as planned, we included age as a covariate for all subsequent analyses.

**Table 2:** Correlations between all key variables.

	*M (SD)*	1	2	3	4	5	6	7	8	9
1. Perceived social support (Time 1)	3.89 (0.87)									
2. Perceived social support (Time 2)	3.77 (1.11)	0.75^***^								
3. Social network	4.73 (1.44)	0.27^*^	0.29^**^							
4. Social constraints	2.09 (0.66)	−0.47^***^	−0.42^***^	−0.24^*^						
5. Depression symptoms (Time 1)	17.12 (11.99)	−0.39^**^	−0.42^***^	−0.23^*^	0.51^***^					
6. Depression symptoms (Time 2)	15.36 (10.61)	−0.48^***^	−0.37^**^	−0.14	0.55^***^	0.75^***^				
7. PTS symptoms (Time 1)	20.52 (16.24)	−0.23^*^	−0.15	0.01	0.37^**^	0.75^***^	0.66^***^			
8. PTS symptoms (Time 2)	17.56 (15.33)	−0.31^**^	−0.15	−0.06	0.40^**^	0.63^***^	0.83^***^	0.69^***^		
9. Injury severity	8.87 (6.49)	−0.02	0.21	0.02	−0.05	−0.14	−0.01	−0.05	0.07	
10. Age	41.54 (12.48)	−0.27^*^	−0.32^**^	−0.13	0.12	0.15	0.01	−0.09	−0.08	−0.07

M, mean; SD, standard deviation; PTS, post-traumatic stress. ***p < 0.001, **p < 0.01, *p < 0.05.

#### Cross-sectional-analyses: Testing for differences by cohort

The results of the independent-samples *t*-tests, followed by the TOST equivalence test procedure for each social support measure suggested that, overall, there were no cohort differences in reported social support on any dimension. Independent-samples *t*-tests showed no statistically significant difference between the pre-COVID-19 (*M* = 3.81, *SD* = 1.00) and COVID-19 (*M* = 3.96, *SD* = 0.94) cohorts in perceived social support at time 1, *t*(82) = −0.74, *p* = 0.462, *d* = 0.16, and results from the TOST procedure indicated that the observed effect size significantly fell within the bounds of *d* = −0.55 and *d* = 0.55, *t*(82) = 1.81, *p* = 0.037 and could be interpreted as equivalent. Moreover, there was no cohort difference (pre-COVID-19: *M* = 4.83, *SD* = 1.48; COVID-19: *M* = 4.62, *SD* = 1.41) in social network size, *t*(82) = 0.68, *p* = 0.499, *d* = 0.15, and this effect size also significantly fell within the equivalence bounds, *t*(82) = −1.86, *p* = 0.034. Finally, there was no significant cohort difference (pre-COVID-19: *M* = 2.10, *SD* = 0.77; COVID-19: *M* = 2.07, *SD* = 0.54) for social constraints, *t*(82) = 0.22, *p* = 0.826, *d* = 0.05, with the observed effect size also falling within the equivalence bounds, *t*(82) = −2.31, *p* = 0.012.

Next, we conducted independent-samples *t*-tests followed by the TOST procedure for each measure of psychological symptoms (depression and PTS) at time 1. Although there was no statistically significant cohort difference for depression symptoms at time 1 (pre-COVID-19: *M* = 15.76, *SD* = 12.57; COVID-19: *M* = 18.48, *SD* = 11.3, *t*(82) = −1.04, *p* = 0.302, the observed effect size (*d* = −0.23, 95% CI [−0.66, 0.20]) was not significantly within the equivalence bounds of *d* = −0.55 and *d* = 0.55, *t*(82) = 1.48, *p* = 0.071, suggesting that null effects did not reflect equivalence, but rather could be explained by other factors, including insufficient statistical power. Next, an independent-samples *t*-test showed no significant cohort difference in PTS symptoms at time 1 (pre-COVID-19: *M* = 19.76, *SD* = 16.86; COVID-19: *M* = 21.29, *SD* = 15.77), *t*(82) = −0.43, *p* = 0.670, and the observed effect size (*d* = 0.09, 95% CI [−0.52, 0.34]) was significantly within the equivalence bounds of *d* = −0.55 and *d* = 0.55, *t*(82) = 2.09, *p* = 0.020, suggesting clear evidence of no difference in PTS symptoms between the cohorts. Thus, there were no differences in symptoms at time 1, but our sample may have been underpowered to detect a true difference in depression at time 1, whereas for PTS symptoms, we could confirm equivalence in cohorts.

#### Longitudinal analyses: Testing for cohort differences in social support and psychological health over time

**Figure 1:**
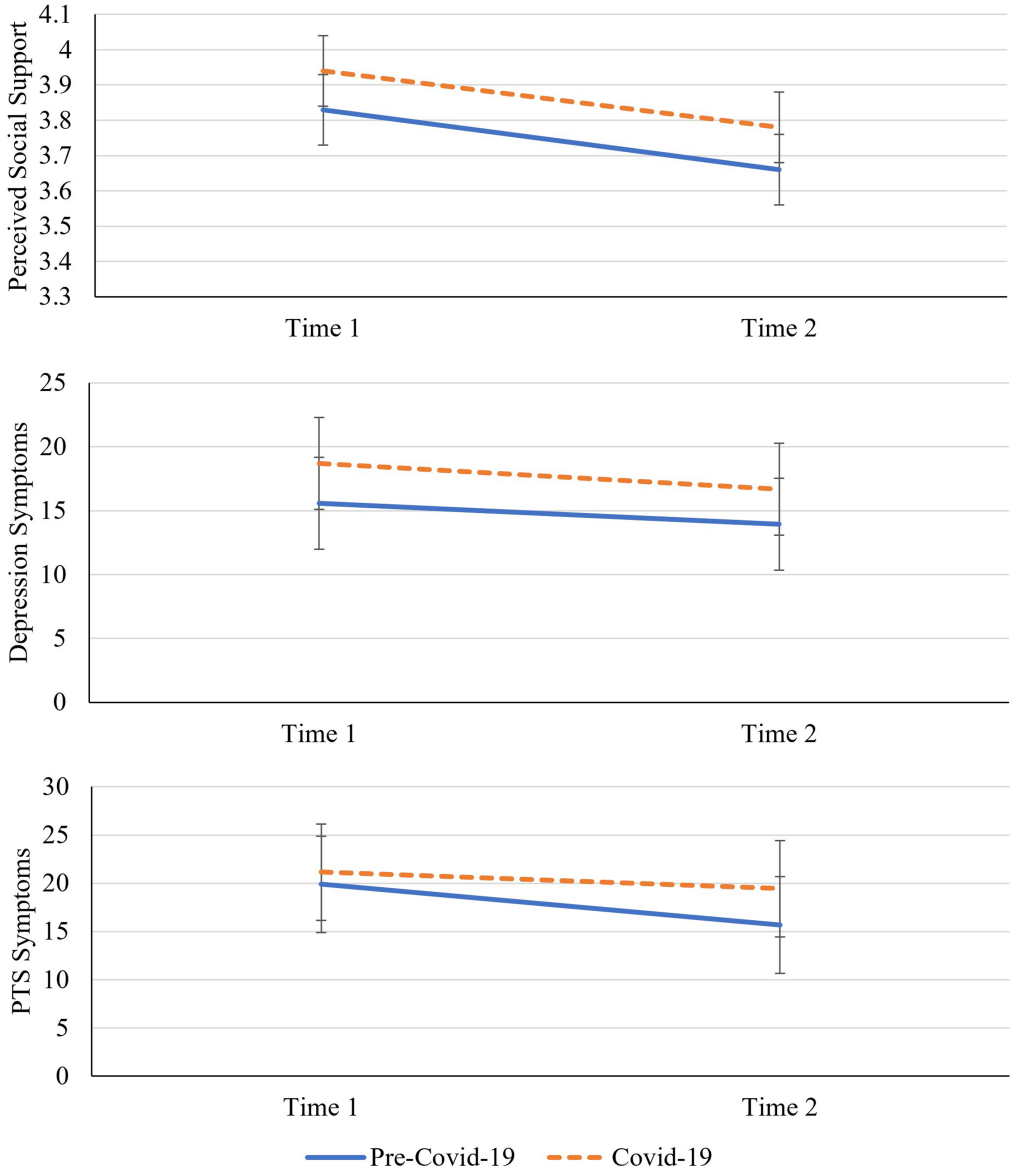
Longitudinal changes in perceived social support, depression and PTS symptoms by cohort. PTS symptoms denotes post-traumatic stress symptoms. Error bars = 95% confidence intervals.

Next, we conducted a repeated measure ANCOVA (covarying age) to evaluate longitudinal changes and cohort differences in perceived social support (times 1 and 2). Although perceived social support decreased across both cohorts from time 1 (*M* = 3.88, *SE* = 0.10) to time 2 (*M* = 3.71, *SE* = 0.12), the change was not statistically significant, *F*(1,80) = 0.29, *p* = 0.593, *μ_p_^2^* = 0.00, and there was no interaction with cohort, *F*(1,80) = 0.17, *p* = 0.685, *μ_p_^2^* = 0.00. Moreover, there was no overall cohort difference in perceived social support, *F*(1,80) = 1.17, *p* = 0.283, *μ_p_^2^* = 0.01. Results were nearly identical with or without age included in the model. We applied the TOST procedure to examine equivalence at time 2 in perceived social support (time 1 was tested above; pre-COVID-19: *M* = 3.63, *SD* = 1.13; COVID-19: *M* = 3.82, *SD* = 1.08) and the observed effect significantly fell within the equivalence bounds (−0.55 to 0.55), *t*(82) = 1.73, *p* = 0.044 suggesting equivalence in perceived social support across time for both cohorts. See Figure 1 for a plot of perceived social support over time.

Next, we tested longitudinal patterns of depression over time, also using a repeated measures ANCOVA (controlling for age). There was no statistically significant change in depression symptoms from time 1 (*M* = 17.12, *SE* = 1.30) to time 2 (*M* = 15.36, *SE* = 1.16), *F*(1,81) = 1.63, *p* = 0.206, *μ_p_^2^* = 0.02, no interaction with cohort, *F*(1,81) = 0.30, *p* = 0.862,, *μ_p_^2^* = 0.00, and no overall cohort difference in depression symptoms, *F*(1,81) = 1.64, *p* = 0.204,, *μ_p_^2^* = 0.02. Again, results were nearly identical when age was removed from the model. Although there were no cohort difference in depression symptoms at time 2 (pre-COVID-19: *M* = 13.98, *SD* = 11.51; COVID-19: *M* = 16.74, *SD* = 9.57), *t*(82) = −1.20, *p* = 0.235, the TOST procedure indicated that the observed effect size (*d* = 0.26, 95% CI[−0.69, 0.17]) was not significantly within the equivalence bounds, *t*(82) = 1.33, *p* = 0.094, suggesting that our sample may have been underpowered to detect a true difference in depression at time 2 (consistent with the effects for time 1). See Figure 1 for a plot of depression symptoms over time.

Lastly, we applied ANCOVA to test PTS symptoms and there was again no statistically significant change in PTS symptoms from time 1 (*M* = 20.52, *SE* = 1.79) to time 2 (*M* = 17.56, *SE* = 1.68), *F*(1,81) = 0.67, *p* = 0.416, *μ_p_^2^* = 0.01. However, when age was removed from the model, the reduction in PTS symptoms from time 1 to time 2 became statistically significant, *F*(1,82) =4.79, *p* = 0.032, *μ_p_^2^* = 0.06. Removing age did not impact any of the other results of this repeated measures ANCOVA. Moreover, there was no interaction with cohort, *F*(1,81) = 0.84, *p* = 0.362, *μ_p_^2^* = 0.01, and no overall cohort difference, *F*(1,81) = 0.61, *p* = 0.436, *μ_p_^2^* = 0.01. Although there were no statistically significant cohort differences in PTS symptoms at time 2 (pre-COVID-19: *M* = 15.56, *SD* = 16.13; COVID-19: *M* = 19.55, *SD* = 14.41), *t*(82) = −1.19, *p* = 0.237, the TOST procedure indicated that the observed effect size (*d* = −0.26, 95% CI [−0.69, 0.17]) was not significantly within the equivalence bounds, *t*(82) = 1.33, *p* = 0.094. Thus, our sample may have been underpowered to detect true differences in PTS at time 2. See Figure 1 for the plotted changes and cohort differences in PTS symptoms.

#### Moderation analyses: Testing the impact of cohort on the process of adjustment

We applied Ordinary Least Squares Regression (OLS) models to examine symptoms reported over time. We first tested longitudinal models with depression (time 2) as the outcome variable and modeled each of the three social support measures (at time 1) as predictors in separate models. Per the Benjamini-Hochberg corrections for tests with depression as the outcome, value of ps less than or equal to 0.003 were interpreted as statistically significant. The first model in this set tested perceived social support at time 1, cohort, and their interaction term as predictors (each with their own step), controlling for age and time 1 depression symptoms in step 1 of the model. Results showed that higher perceived social support at time 1 predicted reduced depression symptoms at time 2 (*B* = −2.88, *p* = 0.001, 95% CI [−4.57, −1.19], *sr^2^* = 0.05), but there was no effect of cohort (*B* = 1.29, *p* = 0.389, 95% CI [−1.67, 4.25]), and no evidence of moderation (*B* = −0.01, *p* = 0.996, 95% CI [−3.10, 3.08]). There was no significant effect of age (*B* = −0.13, *p* = 0.035, 95% CI [−0.25, −0.01], *sr^2^* = 0.02), but depression at time 1 (*B* = 0.58, *p* < 0.001, 95% CI [0.45, 0.72], *sr^2^* = 0.35) significantly contributed to the model. The final model accounted for approximately 62% of the variance in depression at time 2, *F*(5,78) = 25.87, *p* < 0.001, *R^2^* = 0.62. Next, we replaced perceived social support with social network at time 1 in the model. Results showed that only depression symptoms at time 1 significantly predicted depression symptoms at time 2 (*B* = 0.68, *p* < 0.001, 95% CI [0.54, 0.81], *sr^2^* = 0.55). Social network had no significant effect on depression at time 2 (*B* = 0.23, *p* = 0.683, 95% CI [−0.89, 1.35]), and there was no effect of cohort (*B* = 0.76, *p* = 0.633, 95% CI [−2.39, 3.91]), no evidence of moderation (*B* = 1.72, *p* = 0.119, 95% CI [−0.453, 3.90]), and no effect of age (*B* = −0.08, *p* = 0.197, 95% CI [−0.20, 0.54]). The final model accounted for 58% of the variance in depression symptoms, *F*(5,78) = 21.85, *p* < 0.001, *R^2^* = 0.58. Lastly, we ran the model with social constraints at time 1 as the predictor. Higher reported social constraints predicted elevated depression at time 2 (*B* = 3.98, *p* = 0.003, 95% CI [1.38, 6.58], *sr^2^* = 0.05), but there was no effect of cohort (*B* = 1.13, *p* = 0.454, 95% CI [−1.86, 4.12]), and no evidence of moderation (*B* = −1.63, *p* = 0.501, 95% CI [−6.42, 3.17]). There was no effect of age (*B* = −0.09, *p* = 0.127, 95% CI [−0.21, 0.03]), and depression at time 1 predicted higher depression at time 2 (*B* = 0.56, *p* < 0.001, 95% CI [0.41, 0.70], *sr^2^* = 0.28), and the final model accounted for 62% of the variance in depression symptoms, *F*(5,78) = 25.10, *p* < 0.001, *R^2^* = 0.62,. See Supplementary Tables S3–S5 for full regression results for all three models. When age was removed from the above models, the results were nearly identical.

**Figure 2:**
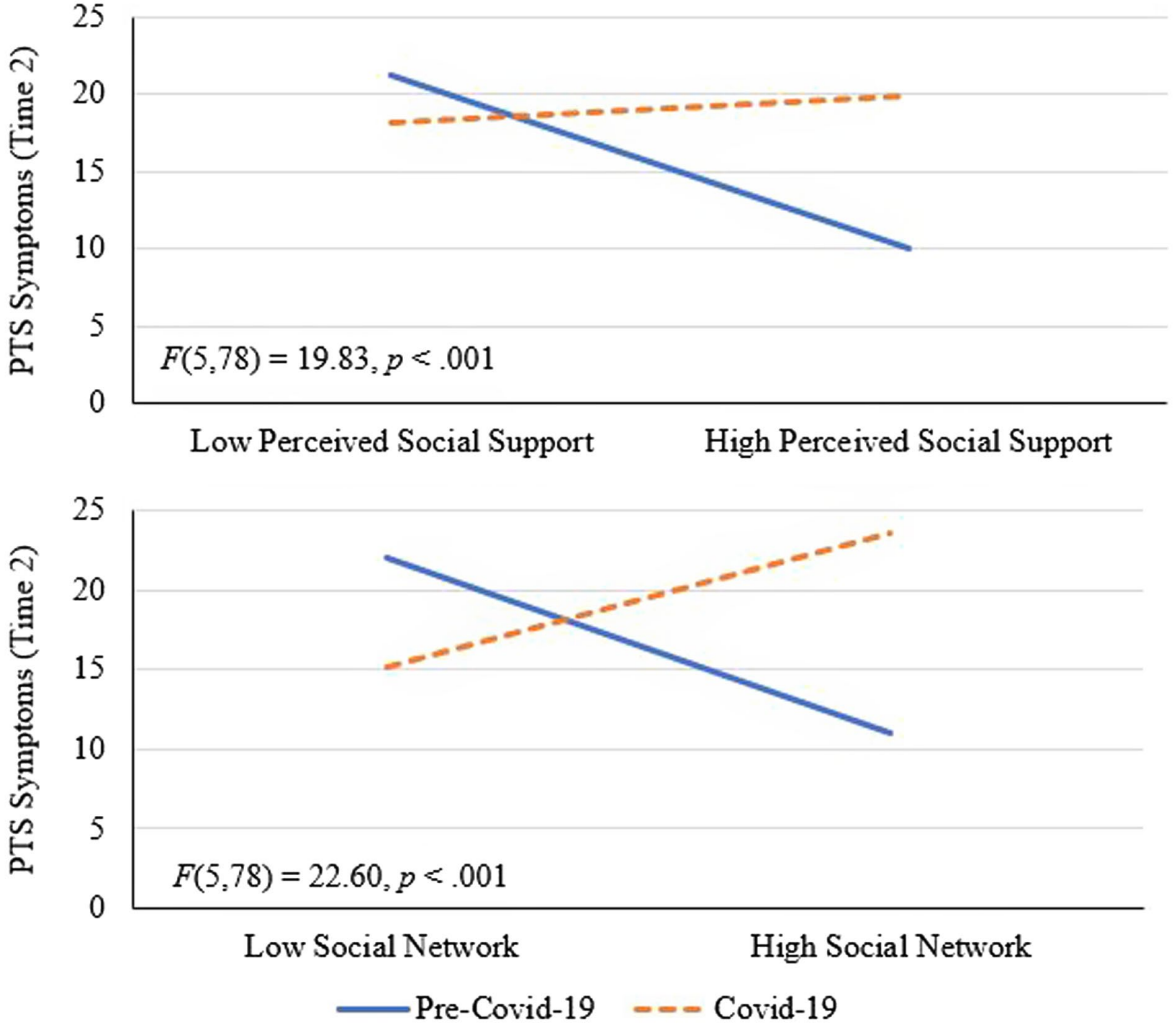
Interactions between perceive social support and cohort and social network and cohort in the prediction of post-traumatic stress symptoms at Time 2. PTS symptoms denotes post-traumatic stress symptoms.

Next, we re-ran the same OLS regression models, but with PTS symptoms at time 2 as the outcome variable. Per the Benjamini-Hochberg corrections, *p*-values less than or equal to.008 were interpreted as statistically significant. After correcting for Type I error, results from the first model (perceived social support as predictor) showed that the negative relationship between perceived social support at time 1 and PTS symptoms at time 2 was non-significant (*B* = −2.88, *p* = 0.034, 95% CI [−5.53, −0.22], *sr^2^* = 0.03). Moreover, there was no effect of cohort (*B* = 3.31, *p* = 0.173, 95% CI [−1.48, 8.09]) or age (*B* = −0.08, *p* = 0.447, 95% CI [−0.28, 0.12]). However, there was clear evidence of moderation by cohort, as a significant cohort by perceived social support (time 1) interaction emerged (*B* = 6.53, *p* = 0.008, 95% CI [1.74, 11.33], *sr^2^* = 0.04), *F*(5,78) = 19.83, *p* < 0.001, *R^2^* = 0.56. Tests of simple slopes showed a significant negative (or protective) association between perceived social support (time 1) and PTS symptoms (time 2) for the pre-COVID-19 cohort (*B* = −5.89, *p* < 0.001, 95% CI [−9.27, −2.51]) but there was no significant slope for the COVID-19 cohort, which suggested an absence of a protective impact of social support in those individuals (*B* = 0.64, *p* = 0.726, 95% CI [−2.99, 4.27]). See Figure 2 for the plotted interaction. Results from the second model (social network at time 1 as predictor) showed no unique effect for social network (*B* = −0.65, *p* = 0.456, 95% CI [−2.36, 1.07]), age (*B* = −0.03, *p* = 0.805, 95% CI [−0.22, 0.18]), nor cohort (*B* = 2.78, *p* = 0.262, 95% CI [−2.13, 7.69]). However, PTS symptoms (time 1) predicted PTS symptoms at time 2 (*B* = 0.65, *p* < 0.001, 95% CI [0.50, 0.80], *sr^2^* = 0.47) and there was again a significant cohort by social network interaction (*B* = 6.83, *p* < 0.001, 95% CI [3.69, 9.96], *sr^2^* = 0.10), *F*(5,78) = 22.60, *p* < 0.001, *R^2^* = 0.59. Tests of simple slopes showed a significant negative relationship between social network and PTS symptoms for the pre-COVID-19 cohort (*B* = −3.85, *p* < 0.001, 95% CI [−5.99, −1.71]), but a significant *positive* relationship for the COVID-19 cohort (*B* = 2.98, *p* = 0.011, 95% CI [0.70, 5.25]). See Figure 2 for the plotted interaction that suggests that a larger social network exerted potentially negative effects (associated with increased PTS symptoms) on adjustment in the COVID-19 cohort. Lastly, results from the third model (social constraints at time 1 as predictor) showed that PTS symptoms at time 1 predicted PTS symptoms at time 2 (*B* = 0.59, *p* < 0.001, 95% CI [0.43, 0.76]), but there were no unique effects for age (*B* = −0.05, *p* = 0.640, 95% CI [−0.24, 0.15]), cohort (*B* = 3.11, *p* = 0.193, 95% CI [−14.49, 0.76]), nor social constraints (*B* = 4.10, *p* = 0.041, 95% CI [0.16, 8.04]). Moreover, in this case, there was no evidence of moderation by cohort (*B* = −6.87, *p* = 0.077, 95% CI [−14.49, 0.76]). The final model accounted for approximately 54% of the variance in PTS symptoms, F(5,78) = 17.97, p < 0.001, *R^2^* = 0.54. See Supplementary Tables S6–S8 for full results of the above three OLS regression models in the Supplementary material. As in the prior analyses, when age was removed from the above three models, the results remained nearly identical.

Finally, we explored whether there was any moderation of perceived social support at time 2 by cohort, when predicting time 2 symptoms. The results were very consistent with the above mentioned findings, suggesting a lasting influence of the context (cohort) on the benefits of social support when predicting PTS symptoms [(*B* = 6.52, *p* = 0.003, 95% CI (2.25, 10.79), *sr^2^* = 0.05), R^2^ = 0.52, *F*(5,78) = 18.85, *p* < 0.001], but no moderation for depression. The full details of these analyses are reported in the Supplementary Tables S9, S10.

#### Exploratory analyses: Further exploring moderation of perceived social support by cohort in predicting post-traumatic stress symptoms (Time 2)

Because PTS and depression symptoms at time 2 were strongly correlated (r = 0.83), we conducted additional analyses to further explore why our results suggested that cohort moderated the relationship between social support and PTS symptoms, but not depression. Specifically, we explored whether the moderation effects were driven by symptoms unique to PTSD (e.g., avoidance and arousal) following compelling evidence from prior research (McGuire et al., 2018). To do this we first computed modified PTS symptom variables by taking the sum of the items from the PCL-5 representing DSM-5 criterion C (avoidance) and criterion E (arousal) symptoms (items 6 and 7 and items 15–20, respectively) consistent with recommendations (Weathers et al., 2013). For this modified PTS symptoms score, scores could range from 0 to 32. Internal consistency was good for both time points (Time 1: α = 0.82; Time 2: α = 0.85). Mean symptoms were 8.56 (*SD* = 6.61) at time 1 and 8.06 (*SD* = 6.68) at time 2 and consistent with the earlier analyses, independent-samples *t*-tests showed no significant cohort difference for either time points (Time 1: *t*(82) = −0.74, *p* = 0.461, *d* = 0.16; Time 2: *t*(82) = −1.46, *p* = 0.147, *d* = 0.32).

Next, we conducted linear OLS regression models using the same approach as the primary longitudinal analyses above, but instead of the full PTS symptoms score, we substituted in the modified PTS indices (time 1 as a covariate, time 2 as the outcome variable). Results from the first model (perceived social support as the predictor) were consistent with the original analysis with the full PTS symptoms scale, such that cohort moderated the relationship between perceived social support and PTS symptoms (*B* = 2.97, *p* = 0.008, 95% CI [0.81, 5.13], *sr^2^* = 0.04), *F*(5,78) = 18.10, *p* < 0.001, *R^2^* = 0.54. Tests of simple slopes were also consistent with the primary analyses, showing that higher perceived social support predicted lower PTS symptoms for the pre-COVID-19 cohort (*B* = −3.00, *p* < 0.001, 95% CI [−4.52, −1.48], but not for the COVID-19 cohort (*B* = −0.03, *p* = 0.804, 95% CI [−1.63, 1.57]). Results from the second model (social network as the predictor) were also consistent with the primary analysis, with a significant cohort by social network interaction (*B* = 2.66, *p* < 0.001, 95% CI [1.21, 4.11], *sr^2^* = 0.08), *F*(5,78) = 17.69, *p* < 0.001, *R^2^* = 0.53, and tests of simple slopes showed that a larger social network predicted lower PTS symptoms for the pre-COVID-19 cohort (*B* = −1.63, *p* = 0.002, 95% CI [−2.63, −0.63]) but not for the COVID-19 cohort (*B* = 1.03, *p* = 0.056, 95% CI [−0.03, 2.08]. The final model included social constraints as the predictor and the results were also consistent with the original analyses, such that there was no moderation by cohort in the prediction of PTS symptoms at time 2 (*B* = −3.00, *p* = 0.088, 95% CI [−6.46, 0.45]), *F*(5,78) = 15.45, *p* < 0.001, *R^2^* = 0.50. See Supplementary Tables S11–S13 for full regression details.

## Discussion

In this investigation, we tested the impact of the COVID-19 pandemic on social support and the process of adjustment following traumatic events in United States adults. Using a matched case–control design, we compared reports of social support and psychological symptoms in United States adults in the first 6 months following traumatic events (e.g., motor vehicle crashes, violence). The two cohorts (pre-COVID-19 and during-COVID-19) were defined by recruitment and data collection date to ensure no overlap for the comparison and were extracted from a larger sample. We carefully matched cohorts based on key characteristics relevant to psychological adjustment (e.g., income, gender). The results suggested that the cohorts were equivalent in reports of social support and psychological symptoms at either the initial stage after the event (approximately 1-month post-event) and 4 months later. Although comparison of symptom reports by cohort were not statistically significant, equivalence tests suggested that differences, particularly for depression, were still possible. Longitudinal analyses of adjustment suggested that, as expected, social support indicators were generally associated with improved adjustment over time (including reduced depression and post-traumatic stress symptoms). However, there was key evidence that cohort, or the specific timing of data collection (pre-versus during-COVID-19) moderated the process of adjustment. Specifically, the results indicated that individuals in the COVID-19 cohort (as compared to the pre-COVID cohort) did not receive as much benefit from social support over time in relation to symptoms of post-traumatic stress. In sum, although generally there was evidence that reports of symptoms and social support were equivalent in participants before or during the COVID-19 pandemic, and that social support largely enhanced psychological adjustment to trauma, there was some evidence suggesting that during the COVID-19 pandemic, social support offered significantly less protection from psychological symptoms to U.S. adults recovering from traumatic events.

The COVID-19 pandemic has been an international crisis, with lock-down and social distancing orders altering the social lives of billions of people leading to what many have called a “loneliness epidemic” (Horigian et al., 2020; Killgore et al., 2020; Okruszek et al., 2020; Tso and Park, 2020). However, consistent with our findings, cross-sectional and longitudinal data have shown little overall change in loneliness after the onset of COVID-19 (Aknin et al., 2022). There has also been evidence that demographic factors have influenced individual variability in adjustment to this stressor. For example, there is clear evidence of sex differences in adjustment, potentially due to differences in parenting roles and the burden of remote schooling on mothers in particular (Connor et al., 2020). Moreover, there is evidence, particularly in the United States, that economic status greatly impacted not only the psychological stress of individuals, but even the risk for the disease (Finch and Hernández Finch, 2020). However, importantly, in our investigation, we found no difference in reports of social support even when holding key factors such as economic status and gender, constant through careful cohort matching. Specifically, our tests of equivalence suggested that three different reports of social support—perceived social support, social network size, and social constraints—were equivalent regardless of whether made prior to, or during, the pandemic. Moreover, there was no evidence of a significant difference in psychological symptoms between the two cohorts at the initial stages of adjustment.

Our analysis of longitudinal adjustment revealed some more complex findings, suggesting that the process of adjustment for adults following trauma, may have been impacted by the pandemic. For example, although our null hypothesis tests of both depression and post-traumatic stress (PTS) symptoms at 5 months post-event were not significant, our equivalence tests indicated that we could not presume that there were no differences between cohorts. Moreover, we did find significant moderation by COVID-19 cohort when predicting PTS symptoms at 5 months, suggesting that social support was less protective during adjustment to trauma during the pandemic, as compared to just prior to the pandemic. There is limited research examining social support during the pandemic in relation to PTS symptoms. One prior investigation of perceived social support during COVID-19 found that higher perceived social support predicted less post-traumatic stress symptoms (Zaken et al., 2022). However, this investigation was cross-sectional and did not test a longitudinal model of adjustment. Importantly, the authors suggested that social support is effective at buffering against some stressors but may not be effective at buffering against the stressors directly related to COVID-19. Our findings support this hypothesis by providing evidence that social support was less effective at reducing post-traumatic stress symptoms during COVID-19 compared to pre-COVID-19.

One explanation may be that although individuals reported the same amount of social support, the quality of that support may have differed. During social distancing orders, many social interactions were moved to virtual settings, which may provide less social reward than in-person interactions (Ferreira et al., 2022; Towner et al., 2022). It is also plausible that other stressors related to COVID-19 reduced the protective effects of social support. Indeed, there is evidence of increased reports of anxiety around opportunities for exposure in social contacts (Etilé and Geoffard, 2022; Saeed et al., 2022), therefore social interactions may have fueled more anxiety compared to pre-COVID-19 interactions. Although our cohorts were matched based on age, sex, education, income, and injury severity, there may be other variables that were influential within the context of COVID-19. Research in the first year of the pandemic has suggested that personal experience with, or proximity to, COVID-19 was a predictor of psychological health (Aknin et al., 2022). Specifically, being personally diagnosed with, or having symptoms of, Covid-19 was associated with increased psychological distress; having a friend or family member with COVID-19 increased reported anxiety; and being around COVID-19 (e.g., healthcare workers) was a risk factor for greater psychological distress (Aknin et al., 2022). Research has also suggested that *how* people spent their time during the pandemic was influential, with activities like gardening, exercising, and reading leading to lower rates of psychological distress and higher rates of life satisfaction (Disabato et al., 2022). Thus, many factors beyond social support contributed to individuals’ adjustment to the pandemic.

One complicated finding from the current investigation was that COVID-19 erased the protective effects of social support against PTS symptoms, but *not* depression symptoms. That is, social support was equally protective against depression symptoms for both cohorts but was less protective against PTS symptoms for the COVID-19 group. This difference could be due to qualitative differences between PTS and depression symptoms. Specifically, PTS symptoms are thought to differ from depression on dimensions of arousal and avoidance (Metzger et al., 2004; American Psychiatric Association, 2013), and past research (though limited) has investigated the differential relationships between social support and different clusters of PTS symptoms (McGuire et al., 2018). Indeed, Joseph et al. (1997) previously presented a theoretical model proposing that social support should impact PTS symptom clusters differently. For example, they suggested that arousal-based symptoms likely benefit from emotion regulatory resources received from positive social interactions, whereas PTS symptoms related to distressing thoughts (e.g., symptoms more closely related to depression) may be affected by others’ interpretations. Importantly, McGuire et al. (2018) investigated the protective effects of social support on depression and PTS symptom clusters following trauma exposure in Hurricane Katrina victims. Results showed protective effects of social support for depression symptoms as well as avoidance-and arousal-based PTS symptoms for individuals who were *not* displaced by the hurricane. However, the protective effects of social support on arousal-and avoidance-based PTS symptoms were *not* evident in victims displaced by the hurricane, whereas the protective effect nonetheless remained for depression. Thus, *both* our and McGuire and colleagues’ findings show that the protective effects of social support on PTS symptoms (but not depression symptoms) are diminished under conditions of higher stress (COVID-19 for the present study, displacement by a hurricane for McGuire and colleagues). Moreover, our exploratory analyses specifying the relationship between social support and arousal-and avoidance-based PTS symptoms further support this pattern of findings. However, more research is needed to delineate the differential impact of social support on PTS and depression symptoms in environmental contexts of high stress.

Importantly, some interesting differences in outcomes emerged depending on which measure of social support was included in the model. Indeed, the three indices of social support have important conceptual differences. For example, perceived social support (or functional social support) is thought to directly buffer against stress, whereas the benefits of a larger social network (or structural social support) are thought to come from a greater sense of belongingness and access to resources (Cohen and Wills, 1985; Kawachi and Berkman, 2001; Stewart et al., 2022). Overall, perceived social support had the most reliable protective effect across analyses, but individuals in the COVID-19 cohort did not appear to receive this benefit when PTS symptoms were the outcome. Further, results indicated that a larger social network may have even been harmful for individuals in the COVID-19 cohort (but protective for the pre-COVID-19 cohort). It is possible that these two findings go hand-in-hand. Given that individuals in the COVID-19 cohort did not benefit from the perceived receipt of social support from close others, having a larger social network could have increased feelings of frustration with less access to close others during the time of their adjustment period. In addition, some, though limited, research has highlighted contexts in which social support is experienced as burdensome (e.g., individuals with chronic illness; Palant and Himmel, 2019). However, this line of research has not been extended to the context of the COVID-19 pandemic, and any additional commentary would be conjecture. Rather, our findings highlight a need for further investigation into potential nuanced effects of social support in the context of normal adjustment to PTSD-qualifying events during a broader stressful context such as the COVID-19 pandemic. In particular, it seems highly relevant that research consider broader contextual processes as well as multiple operationalizations of social support in future investigations of this kind.

This study provides compelling evidence that social support was equivalent regardless of timing in relation to the COVID-19 pandemic. A major strength of our investigation is the case–control design in which cohorts were rigorously matched based on age, gender, race/ethnicity, education, reported income, and injury severity status. We included multiple indices for comparison and operationalized social support in three ways: perceived social support, social network size, and social constraints within important relationships. Given the relative consistency in our findings across operationalizations of social support, we can have more confidence in our results. We were also able to model symptoms longitudinally, with symptoms reported at two time points approximately 4 months apart, in a high-risk sample recruited shortly after traumatic events and injury.

Despite these key strengths, there were a number of limitations. The rigor of the case–control matched design did, unfortunately, limit the overall sample size included, and there was some indication that null findings in symptom comparisons by cohort at 5 months may have been underpowered. Thus, replication studies will be essential to establish the reliability of our results. Importantly, future research should aim for samples with enough power to model multiple independent and dependent variables within a single structural equation model to better control for shared variance between depression and post-traumatic stress symptoms and social support predictors. Moreover, we were not powered to test 3-way interactions. In particular, we were not able to consider whether other factors known to influence risk for symptoms, such as gender, also moderated the interaction between social support and cohort in the prediction of symptoms. Further, we were not able to measure different forms of social interaction (e.g., in-person versus virtual), which may have differed by cohort. One limitation related to the matching of cohorts was that we did not have injury severity scores (ISS) for 6 participants—5 from the COVID-19 cohort—which may have impacted the results of the matching process. Finally, our analyses did not consider other dimensions of psychological adjustment, namely indices of psychological well-being or daily functioning, both of which would be relevant to this question. Despite these limitations, the rigor of the matched cohort longitudinal design makes the findings novel and impactful, and certainly warrants replication and expansion.

In sum, this investigation aimed to examine how social support may have deviated in form and/or function during the COVID-19 pandemic in United States adults recovering from potentially traumatic events. By applying a matched case–control design, we were able to test if reports of social support differed between patients who either experienced trauma before or during the pandemic. The matched design allowed us to isolate effects that could otherwise have been accounted for by key factors known to impact adjustment during the pandemic, namely economic status and gender. Moreover, we were able to test how social support operated in the service of psychological adjustment and found some evidence that social support, although reported at equivalent levels across cohorts, was less protective when traumatic events occurred within the context of the COVID-19 pandemic when compared to pre-pandemic. In sum, we found compelling evidence that the process of psychological adjustment to trauma may have shifted in part, due to pandemic-related impacts. These findings are novel and impactful, as they shed light on the specific impacts of the COVID-19 pandemic on psychological processes, but also test the role of broader, perhaps more chronic, environmental stressors on adjustment to an acute traumatic event.

## Data availability statement

The datasets presented in this study can be found in online repositories. The names of the repository/repositories and accession number(s) can be found in the article/Supplementary material.

## Ethics statement

The studies involving human participants were reviewed and approved by Kent State University Institutional Review Board. The patients/participants provided their written informed consent to participate in this study.

## Author contributions

BM, EG, BB, and KC contributed to the conceptualization and methodology, and wrote the original draft. BM and KC did the formal data analyses. KC, RG, FM, AM, JG, and DD contributed to the acquisition of resources and funding. BM did the data curation and created the tables and figures. KC oversaw supervision and project administration. All authors contributed to the investigation, review, and editing of the manuscript.

## Funding

This study was supported by a National Institutes of Health grant number (no. R01 MH113622) to KC.

## Conflict of interest

The author declares that the research was conducted in the absence of any commercial or financial relationships that could be construed as a potential conflict of interest.

## Publisher’s note

All claims expressed in this article are solely those of the authors and do not necessarily represent those of their affiliated organizations, or those of the publisher, the editors and the reviewers. Any product that may be evaluated in this article, or claim that may be made by its manufacturer, is not guaranteed or endorsed by the publisher.
