# Supplementary material for: Were there losses in social support during the pandemic? Testing the impact of COVID-19 on psychological adjustment to trauma in United States adults
Source: Front Psychol. 2022 Dec 22;13:1061621. doi: 10.3389/fpsyg.2022.1061621 (PMC9813403; doi:10.3389/fpsyg.2022.1061621)
Supplement: Supplementary file 1 [file Table_1.docx]

**Supplemental Materials For:**

Were there losses in social support during the pandemic?

Testing the impact of COVID-19 on psychological adjustment to trauma in US adults.

The data and code analyzed for this study can be found at the following (anonymized) link: <https://osf.io/n89fx/?view_only=3633c9b5f4d247b5aab89fe2f937cba1>

1. **Results of OLS Regression Analyses with Perceived Social Support (Time 2) as the primary predictor of symptoms (depression and PTS symptoms) at Time 2 (p. 3-4).**
2. **Table S1: *Regression Results with Perceives Social Support (Time 1) Predicting Depression Symptoms (Time 2).* (p. 5).**
3. **Table S2: *Regression Results with Social Network Predicting Depression Symptoms (Time 2).* (p. 6).**
4. **Table S3: *Regression Results with Social Constraints Predicting Depression Symptoms (Time 2).* (p. 7).**
5. **Table S4: *Regression Results with Perceives Social Support (Time 1) Predicting PTS Symptoms (Time 2).* (p. 8).**
6. **Table S5: *Regression Results with Social Network Predicting PTS Symptoms (Time 2).* (p. 9).**
7. **Table S6: *Regression Results with Social Constraints Predicting PTS Symptoms (Time 2).* (p. 10).**
8. **Table S7: *Regression Results with Perceived Social Support (Time 2) Predicting Depression Symptoms (Time 2).* (p. 11).**
9. **Table S8: *Regression Results with Perceived Social Support (Time 2) Predicting PTS Symptoms (Time 2).* (p. 12).**
10. **Table S9: *Regression Results with Perceived Social Support (Time 1) Predicting Avoidance- and Arousal-Based PTS Symptoms (Time 2).* (p. 13).**
11. **Table S10: *Regression Results with Social Network Predicting Avoidance- and Arousal-Based PTS Symptoms (Time 2).* (p. 14).**
12. **Table S11: *Regression Results with Social Constraints Predicting Avoidance- and Arousal-Based PTS Symptoms (Time 2).* (p. 15).**

**Supplemental Moderation Analyses: Testing the Impact of Cohort on the Relationship between Time 2 Perceived Social Support and Time 2 Symptoms.**

We ran similar models as our primary analyses reported in the main text of the manuscript, but with perceived social support (PSS) at *time 2* as the primary predictor of symptoms at time 2 (depression or PTS symptoms), and we investigated moderation by cohort while controlling for age and symptoms at time 1. Results of the first model showed that higher depression symptoms at time 1 predicted higher depression symptoms at time 2 (*B* = 0.63, *p* < .001, 95% CI [0.49, 0.77], *sr^2^* = .40) but there was no unique effect of perceived social support at time 2 (*B* = -1.14, *p* = 292, 95% CI [-2.76, 4.11]). Moreover, there were no unique effects of age (*B* = -0.11, *p* = .092, 95% CI [-0.24, 0.02]), cohort (*B* = 0.98, *p* = .535, 95% CI [-2.15, 4.11]), and there was no moderation of time 2 perceived social support by cohort (*B* = 1.26, *p* = .884, 95% CI [-1.57, 4.09]), *R^2^* = .58, *F*(5,78) = 21.90, *p* < .001. See Table S7 for results of the full model. Lastly, we replaced depression symptoms with PTS symptoms as the outcome variable and re-ran the model. Again, results showed that higher PTS symptoms at time 1 predicted higher symptoms at time 2 (*B* = 0.64, *p* < .001, 95% CI [0.48, 0.79], *sr^2^* = .44), but perceived social support at time 2 had no unique effect on symptoms at time 2 (*B* = -0.91, *p* = .450, 95% CI [-3.29, 1.47]), and there were no unique effects for age (*B* = -0.04, *p* = .698, 95% CI [-0.25, 0.17]) or cohort (*B* = 3.07, *p* = .216, 95% CI [-1.83, 7.97]). However, there was a significant cohort by perceived social support (time 2) interaction (*B* = 6.52, *p* = .003, 95% CI [2.25, 10.79], *sr^2^* = .05), *R^2^* = .52, *F*(5,78) = 18.85, *p* < .001. Results of the tests of simple slopes were consistent with the longitudinal model reported in our primary analyses. Perceived social support at time 2 predicted lower PTS symptoms at time 2 for the Pre-Covid-19 cohort (*B* = -3.85, *p* = .012, 95% CI [-6.82, -0.88]), but this relationship was non-significant for the Covid-19 cohort (*B* = 2.68, *p* = .106, 95% CI [-0.58, 5.93]). See Table S8 for results of the full model.

| Table S1: *Regression Results with Perceives Social Support (Time 1) Predicting Depression Symptoms (Time 2).* | | | | | | | | | |
| --- | --- | --- | --- | --- | --- | --- | --- | --- | --- |
|  |  | *B* | *SE* | *β* | p-value | 95% CI | | *sr^2^* | *R^2^* |
|  |  |  |  |  |  | Lower | Upper |  |  |
| Step 1 | *F*(2,81) = 53.28, *p* < .001 | | |  |  |  |  |  | .57 |
|  | Age | -0.09 | 0.06 | -0.11 | .155 | -0.22 | 0.03 | .01 |  |
|  | Depression Symptoms (Time 1) | 0.67 | 0.07 | 0.76 | <.001 | 0.54 | 0.80 | .57 |  |
| Step 2 | *F*(3,80) = 43.56, *p* < .001 | | |  |  |  |  |  | .62 |
|  | Age | -0.14 | 0.06 | -0.16 | .028 | -0.26 | -0.02 | .02 |  |
|  | Depression Symptoms (Time 1) | 0.59 | 0.07 | 0.67 | <.001 | 0.46 | 0.73 | .38 |  |
|  | Perceived Social Support (Time 1) | -2.79 | 0.84 | -0.26 | .001 | -4.47 | -1.12 | .05 |  |
| Step 3 | *F*(4,79) = 32.76, *p* < .001 | | |  |  |  |  |  | .62 |
|  | Age | -0.13 | 0.06 | -0.15 | .035 | -0.25 | -0.01 | .02 |  |
|  | Depression Symptoms (Time 1) | 0.58 | 0.07 | 0.66 | <.001 | 0.45 | 0.72 | .36 |  |
|  | Perceived Social Support (Time 1) | -2.88 | 0.85 | -0.26 | .001 | -4.57 | -1.19 | .05 |  |
|  | Cohort | 1.29 | 1.49 | 0.06 | .389 | -1.67 | 4.25 | .00 |  |
| Step 4 | *F*(5,78) = 25.87, *p* < .001 | | |  |  |  |  |  | .62 |
|  | Age | -0.13 | 0.06 | -0.15 | .038 | -0.25 | -0.01 | .02 |  |
|  | Depression Symptoms (Time 1) | 0.58 | 0.07 | 0.66 | <.001 | 0.45 | 0.72 | .35 |  |
|  | Perceived Social Support (Time 1) | -2.88 | 0.86 | -0.26 | .001 | -4.58 | -1.17 | .05 |  |
|  | Cohort | 1.29 | 1.50 | 0.06 | .392 | -1.69 | 4.27 | .00 |  |
|  | Cohort *Perceived Social Support (Time 1) | -0.01 | 1.55 | 0.00 | .996 | -3.10 | 3.08 | .00 |  |
| *Note*: *B* = unstandardized beta, *SE* = standard error, *β* = standardized beta, sr2 = semi-partial correlation. | | | | | | | | | |

| Table S2: *Regression Results with Social Network predicting Depression Symptoms (Time 2)* | | | | | | | | |  |
| --- | --- | --- | --- | --- | --- | --- | --- | --- | --- |
|  |  | *B* | *SE* | *β* | p-value | 95% CI | | *sr^2^* | *R^2^* |
|  |  |  |  |  |  | Lower | Upper |  |  |
| Step 1 | *F*(2,81) = 53.28, *p* < .001 | | |  |  |  |  |  | .57 |
|  | Age | -0.09 | 0.06 | -0.11 | .155 | -0.22 | 0.03 | .01 |  |
|  | Depression Symptoms (Time 1) | 0.67 | 0.07 | 0.76 | <.001 | 0.54 | 0.80 | .57 |  |
| Step 2 | *F*(3,80) = 35.20, *p* < .001 | | |  |  |  |  |  | .57 |
|  | Age | -0.09 | 0.06 | -0.10 | .171 | -0.21 | 0.04 | .01 |  |
|  | Depression Symptoms (Time 1) | 0.68 | 0.07 | 0.77 | <.001 | 0.55 | 0.81 | .55 |  |
|  | Social Network | 0.21 | 0.56 | 0.03 | .703 | -0.90 | 1.32 | .00 |  |
| Step 3 | *F*(4,79) = 26.20, *p* < .001 | | |  |  |  |  |  | .57 |
|  | Age | -0.08 | 0.06 | -0.10 | .197 | -0.21 | 0.04 | .01 |  |
|  | Depression Symptoms (Time 1) | 0.68 | 0.07 | 0.76 | <.001 | 0.54 | 0.81 | .54 |  |
|  | Social Network | 0.23 | 0.56 | 0.03 | .683 | -0.89 | 1.35 | .00 |  |
|  | Cohort | 0.76 | 1.58 | 0.04 | .633 | -2.39 | 3.91 | .00 |  |
| Step 4 | *F*(5,78) = 21.85, *p* < .001 | | |  |  |  |  |  | .58 |
|  | Age | -0.07 | 0.06 | -0.09 | .252 | -0.20 | 0.05 | .01 |  |
|  | Depression Symptoms (Time 1) | 0.69 | 0.07 | 0.78 | <.001 | 0.55 | 0.82 | .55 |  |
|  | Social Network | 0.30 | 0.56 | 0.04 | .588 | -0.81 | 1.42 | .00 |  |
|  | Cohort | 0.77 | 1.57 | 0.04 | .626 | -2.35 | 3.89 | .00 |  |
|  | Cohort *Social Network | 1.72 | 1.09 | 0.12 | .119 | -0.45 | 3.90 | .01 |  |
| *Note*: *B* = unstandardized beta, *SE* = standard error, *β* = standardized beta, sr^2^ = semi-partial correlation. | | | | | | | | | |

| Table S3: *Regression Results with Social Constraints Predicting Depression Symptoms (Time 2).* | | | | | | | | | |
| --- | --- | --- | --- | --- | --- | --- | --- | --- | --- |
|  |  | *B* | *SE* | *β* | *p-value* | *95% CI* | | *sr^2^* | *R^2^* |
|  |  |  |  |  |  | Lower | Upper |  |  |
| Step 1 | *F*(2,81) = 53.28, *p* < .001 | | |  |  |  |  |  | .57 |
|  | Age | -0.09 | 0.06 | -0.11 | .155 | -0.22 | 0.03 | .01 |  |
|  | Depression Symptoms (Time 1) | 0.67 | 0.07 | 0.76 | <.001 | 0.54 | 0.80 | .57 |  |
| Step 2 | *F*(3,80) = 42.01, *p* < .001 | | |  |  |  |  |  | .61 |
|  | Age | -0.10 | 0.06 | -0.12 | .103 | -0.22 | 0.02 | .01 |  |
|  | Depression Symptoms (Time 1) | 0.57 | 0.07 | 0.64 | <.001 | 0.42 | 0.71 | .30 |  |
|  | Social Constraints | 3.89 | 1.30 | 0.24 | .004 | 1.31 | 6.48 | .04 |  |
| Step 3 | *F*(4,79) = 31.48, *p* < .001 | | |  |  |  |  |  | .61 |
|  | Age | -0.09 | 0.06 | -0.11 | .127 | -0.21 | 0.03 | .01 |  |
|  | Depression Symptoms (Time 1) | 0.56 | 0.07 | 0.63 | <.001 | 0.41 | 0.70 | .29 |  |
|  | Social Constraints | 3.98 | 1.31 | 0.25 | .003 | 1.38 | 6.58 | .05 |  |
|  | Cohort | 1.13 | 1.50 | 0.05 | .454 | -1.86 | 4.12 | .00 |  |
| Step 4 | *F*(5,78) = 25.10, *p* < .001 | | |  |  |  |  |  | .62 |
|  | Age | -0.09 | 0.06 | -0.10 | .149 | -0.21 | 0.03 | .01 |  |
|  | Depression Symptoms (Time 1) | 0.56 | 0.07 | 0.63 | <.001 | 0.41 | 0.70 | .28 |  |
|  | Social Constraints | 3.72 | 1.37 | 0.23 | .008 | 1.00 | 6.44 | .04 |  |
|  | Cohort | 1.14 | 1.51 | 0.05 | .452 | -1.86 | 4.14 | .00 |  |
|  | Cohort *Social Constraints | -1.63 | 2.41 | -0.05 | .501 | -6.42 | 3.17 | .00 |  |
| *Note*: *B* = unstandardized beta, *SE* = standard error, *β* = standardized beta, sr^2^ = semi-partial correlation. | | | | | | | | | |

| Table S4: *Regression Results with Perceived Social Support (Time 1) Predicting PTS Symptoms (Time 2).* | | | | | | | | | | |
| --- | --- | --- | --- | --- | --- | --- | --- | --- | --- | --- |
|  |  | | *B* | *SE* | *β* | *p-value* | *95% CI* | | *sr^2^* | *R^2^* |
|  |  |  | |  |  |  | Lower | Upper |  |  |
| Step 1 | *F*(2,81) = 37.46, *p* < .001 | | | |  |  |  |  |  | .48 |
|  | Age | -0.03 | | 0.10 | -0.02 | .789 | -0.22 | 0.17 | .00 |  |
|  | PTS Symptoms (Time 1) | 0.65 | | 0.08 | 0.69 | <.001 | 0.50 | 0.80 | .47 |  |
| Step 2 | *F*(3,80) = 27.37, *p* < .001 | | | |  |  |  |  |  | .51 |
|  | Age | -0.09 | | 0.10 | -0.07 | .388 | -0.29 | 0.11 | .00 |  |
|  | PTS Symptoms (Time 1) | 0.61 | | 0.08 | 0.65 | <.001 | 0.46 | 0.76 | .38 |  |
|  | Perceived Social Support (Time 1) | -2.75 | | 1.34 | -0.17 | .043 | -5.41 | -0.09 | .03 |  |
| Step 3 | *F*(4,79) = 21.23, *p* < .001 | | | |  |  |  |  |  | .52 |
|  | Age | -0.08 | | 0.10 | -0.06 | .447 | -0.28 | 0.12 | .00 |  |
|  | PTS Symptoms (Time 1) | 0.60 | | 0.08 | 0.64 | <.001 | 0.45 | 0.76 | .38 |  |
|  | Perceived Social Support (Time 1) | -2.87 | | 1.33 | -0.18 | .034 | -5.53 | -0.22 | .03 |  |
|  | Cohort | 3.31 | | 2.40 | 0.11 | .173 | -1.48 | 8.09 | .01 |  |
| Step 4 | *F*(5,78) = 19.83, *p* < .001 | | | |  |  |  |  |  | .56 |
|  | Age | -0.05 | | 0.10 | -0.04 | .623 | -0.24 | 0.15 | .00 |  |
|  | PTS Symptoms (Time 1) | 0.60 | | 0.07 | 0.63 | <.001 | 0.45 | 0.74 | .37 |  |
|  | Perceived Social Support (Time 1) | -2.63 | | 1.29 | -0.17 | .045 | -5.19 | -0.07 | .02 |  |
|  | Cohort | 3.35 | | 2.31 | 0.11 | .151 | -1.25 | 7.95 | .01 |  |
|  | Cohort *Percieved Social Support (Time 1) | 6.53 | | 2.41 | 0.21 | .008 | 1.74 | 11.33 | .04 |  |
| *Note*: PTS Symptoms = Post-traumatic stress symptoms, *B* = unstandardized beta, *SE* = standard error, *β* = standardized beta, sr^2^ = semi-partial correlation. | | | | | | | | | | |

| Table S5: *Regression Results with Social Network Predicting PTS Symptoms (Time 2).* | | | | | | | | | |
| --- | --- | --- | --- | --- | --- | --- | --- | --- | --- |
|  |  | *B* | *SE* | *β* | *p-value* | *95% CI* | | *sr^2^* | *R^2^* |
|  |  |  |  |  |  | Lower | Upper |  |  |
| Step 1 | *F*(2,81) = 37.46, *p* < .001 | | |  |  |  |  |  | .48 |
|  | Age | -0.03 | 0.10 | -0.02 | .789 | -0.22 | 0.17 | .00 |  |
|  | PTS Symptoms (Time 1) | 0.65 | 0.08 | 0.69 | <.001 | 0.50 | 0.80 | .47 |  |
| Step 2 | *F*(3,80) = 25.13, *p* < .001 | | |  |  |  |  |  | .49 |
|  | Age | -0.04 | 0.10 | -0.03 | .708 | -0.24 | 0.16 | .00 |  |
|  | PTS Symptoms (Time 1) | 0.65 | 0.08 | 0.69 | <.001 | 0.50 | 0.80 | .47 |  |
|  | Social Network | -0.73 | 0.86 | -0.07 | .397 | -2.44 | 0.98 | .00 |  |
| Step 3 | *F*(4,79) = 19.23, *p* < .001 | | |  |  |  |  |  | .49 |
|  | Age | -0.02 | 0.10 | -0.02 | .805 | -0.22 | 0.17 | .00 |  |
|  | PTS Symptoms (Time 1) | 0.65 | 0.08 | 0.69 | <.001 | 0.50 | 0.80 | .47 |  |
|  | Social Network | -0.65 | 0.86 | -0.06 | .456 | -2.36 | 1.07 | .00 |  |
|  | Cohort | 2.78 | 2.47 | 0.09 | .262 | -2.13 | 7.69 | .01 |  |
| Step 4 | *F*(5,78) = 22.60, *p* < .001 | | |  |  |  |  |  | .59 |
|  | Age | 0.03 | 0.09 | 0.02 | .781 | -0.16 | 0.21 | .00 |  |
|  | PTS Symptoms (Time 1) | 0.70 | 0.07 | 0.74 | <.001 | 0.56 | 0.84 | .53 |  |
|  | Social Network | -0.43 | 0.78 | -0.04 | .579 | -1.99 | 1.12 | .00 |  |
|  | Cohort | 2.88 | 2.23 | 0.09 | .199 | -1.55 | 7.32 | .01 |  |
|  | Cohort *Social Network | 6.83 | 1.58 | 0.32 | <.001 | 3.69 | 9.96 | .10 |  |
| *Note*: PTS Symptoms = Post-traumatic stress symptoms, *B* = unstandardized beta, *SE* = standard error, *β* = standardized beta, sr^2^ = semi-partial correlation. | | | | | | | | | |

| Table S6: *Regression Results with Social Constraints Predicting PTS Symptoms (Time 2).* | | | | | | | | | |
| --- | --- | --- | --- | --- | --- | --- | --- | --- | --- |
|  |  | *B* | *SE* | *β* | *p-value* | *95% CI* | | *sr^2^* | *R^2^* |
|  |  |  |  |  |  | Lower | Upper |  |  |
| Step 1 | *F*(2,81) = 37.46, *p* < .001 | | |  |  |  |  |  | .48 |
|  | Age | -0.03 | 0.10 | -0.02 | .789 | -0.22 | 0.17 | .00 |  |
|  | PTS Symptoms (Time 1) | 0.65 | 0.08 | 0.69 | <.001 | 0.50 | 0.80 | .47 |  |
| Step 2 | *F*(3,80) = 27.30, *p* < .001 | | |  |  |  |  |  | .51 |
|  | Age | -0.06 | 0.10 | -0.05 | .555 | -0.25 | 0.14 | .00 |  |
|  | PTS Symptoms (Time 1) | 0.59 | 0.08 | 0.63 | <.001 | 0.43 | 0.75 | .33 |  |
|  | Social Constraints | 4.03 | 1.99 | 0.17 | .046 | 0.08 | 7.98 | .03 |  |
| Step 3 | *F*(4,79) = 21.06, *p* < .001 | | |  |  |  |  |  | .52 |
|  | Age | -0.05 | 0.10 | -0.04 | .640 | -0.24 | 0.15 | .00 |  |
|  | PTS Symptoms (Time 1) | 0.59 | 0.08 | 0.62 | <.001 | 0.43 | 0.75 | .33 |  |
|  | Social Constraints | 4.10 | 1.98 | 0.18 | .041 | 0.16 | 8.04 | .03 |  |
|  | Cohort | 3.09 | 2.40 | 0.10 | .201 | -1.69 | 7.88 | .01 |  |
| Step 4 | *F*(5,78) = 17.97, *p* < .001 | | |  |  |  |  |  | .54 |
|  | Age | -0.03 | 0.10 | -0.02 | .764 | -0.22 | 0.16 | .00 |  |
|  | PTS Symptoms (Time 1) | 0.58 | 0.08 | 0.61 | <.001 | 0.42 | 0.74 | .32 |  |
|  | Social Constraints | 2.97 | 2.05 | 0.13 | .152 | -1.12 | 7.05 | .01 |  |
|  | Cohort | 3.11 | 2.37 | 0.10 | .193 | -1.60 | 7.83 | .01 |  |
|  | Cohort *Social Constraints | -6.87 | 3.83 | -0.15 | .077 | -14.49 | 0.76 | .02 |  |
| *Note*: PTS Symptoms = Post-traumatic stress symptoms, *B* = unstandardized beta, *SE* = standard error, *β* = standardized beta, sr^2^ = semi-partial correlation. | | | | | | | | | |

| Table S7: *Regression Results with Perceives Social Support (Time 2) Predicting Depression Symptoms (time 2).* | | | | | | | | | |
| --- | --- | --- | --- | --- | --- | --- | --- | --- | --- |
|  |  | *B* | *SE* | *β* | *p-value* | *95% CI* | | *sr^2^* | *R^2^* |
|  |  |  |  |  |  | Lower | Upper |  |  |
| Step 1 | *F*(2,81) = 53.28, *p* < .001 | | |  |  |  |  |  | .56 |
|  | Age | -0.09 | 0.06 | -0.11 | .155 | -0.22 | 0.03 | .01 |  |
|  | Depression Symptoms (Time 1) | 0.67 | 0.07 | 0.76 | <.001 | 0.54 | 0.80 | .57 |  |
| Step 2 | *F*(3,80) = 36.49, *p* < .001 | | |  |  |  |  |  | .56 |
|  | Age | -0.12 | 0.07 | -0.14 | .080 | -0.25 | 0.01 | .02 |  |
|  | Depression Symptoms (Time 1) | 0.64 | 0.07 | 0.72 | <.001 | 0.50 | 0.78 | .43 |  |
|  | Perceived Social Support (*Time 2*) | -1.08 | 0.80 | -0.11 | .181 | -2.68 | 0.51 | .01 |  |
| Step 3 | *F*(4,79) = 27.25, *p* < .001 | | |  |  |  |  |  | .56 |
|  | Age | -0.11 | 0.07 | -0.13 | .092 | -0.24 | 0.02 | .02 |  |
|  | Depression Symptoms (Time 1) | 0.63 | 0.07 | 0.71 | <.001 | 0.49 | 0.77 | .40 |  |
|  | Perceived Social Support (*Time 2*) | -1.14 | 0.81 | -0.12 | .163 | -2.76 | 0.47 | .01 |  |
|  | Cohort | 0.98 | 1.57 | 0.05 | .535 | -2.15 | 4.11 | .00 |  |
| Step 4 | *F*(5,78) = 21.90, *p* < .001 | | |  |  |  |  |  | .56 |
|  | Age | -0.10 | 0.07 | -0.12 | .122 | -0.24 | 0.03 | .01 |  |
|  | Depression Symptoms (Time 1) | 0.63 | 0.07 | 0.71 | <.001 | 0.49 | 0.77 | .40 |  |
|  | Perceived Social Support (*Time 2*) | -1.08 | 0.81 | -0.11 | .187 | -2.70 | 0.54 | .01 |  |
|  | Cohort | 0.99 | 1.57 | 0.05 | .531 | -2.14 | 4.12 | .00 |  |
|  | Cohort *Perceived Social Support (*Time 2*) | 1.26 | 1.42 | 0.07 | .379 | -1.57 | 4.09 | .00 |  |
| *Note*: *B* = unstandardized beta, *SE* = standard error, *β* = standardized beta. | | | | | | | |  |  |

| Table S8: *Regression Results with Perceives Social Support (Time 2) Predicting PTS Symptoms (Time 2).* | | | | | | | | | |
| --- | --- | --- | --- | --- | --- | --- | --- | --- | --- |
|  |  | *B* | *SE* | *β* | *p-value* | *95% CI* | | *sr^2^* | *R^2^* |
|  |  |  |  |  |  | Lower | Upper |  |  |
| Step 1 | *F*(2,81) = 37.46, *p* < .001 | | |  |  |  |  |  | 0.48 |
|  | Age | -0.03 | 0.10 | -0.02 | .789 | -0.22 | 0.17 | .00 |  |
|  | PTS Symptoms (Time 1) | 0.65 | 0.08 | 0.69 | <.001 | 0.50 | 0.80 | .47 |  |
| Step 2 | *F*(3,80) = 24.96, *p* < .001 | | |  |  |  |  |  | 0.48 |
|  | Age | -0.05 | 0.11 | -0.04 | .631 | -0.26 | 0.16 | .00 |  |
|  | PTS Symptoms (Time 1) | 0.64 | 0.08 | 0.68 | <.001 | 0.49 | 0.80 | .44 |  |
|  | Perceived Social Support (*Time 2*) | -0.81 | 1.20 | -0.06 | .500 | -3.19 | 1.57 | .00 |  |
| Step 3 | *F*(4,79) = 19.24, *p* < .001 | | |  |  |  |  |  | 0.49 |
|  | Age | -0.04 | 0.11 | -0.03 | .698 | -0.25 | 0.17 | .00 |  |
|  | PTS Symptoms (Time 1) | 0.64 | 0.08 | 0.68 | <.001 | 0.48 | 0.79 | .44 |  |
|  | Perceived Social Support (*Time 2*) | -0.91 | 1.19 | -0.07 | .450 | -3.29 | 1.47 | .00 |  |
|  | Cohort | 3.07 | 2.46 | 0.10 | .216 | -1.83 | 7.97 | .01 |  |
| Step 4 | *F*(5,78) = 18.85, *p* < .001 | | |  |  |  |  |  | 0.55 |
|  | Age | 0.00 | 0.10 | 0.00 | .974 | -0.20 | 0.20 | .00 |  |
|  | PTS Symptoms (Time 1) | 0.65 | 0.07 | 0.68 | <.001 | 0.50 | 0.79 | .45 |  |
|  | Perceived Social Support (*Time 2*) | -0.59 | 1.14 | -0.04 | .609 | -2.86 | 1.69 | .00 |  |
|  | Cohort | 3.11 | 2.34 | 0.10 | .188 | -1.55 | 7.78 | .01 |  |
|  | Cohort *Perceived Social Support (*Time 2*) | 6.52 | 2.14 | 0.23 | .003 | 2.25 | 10.79 | .05 |  |
| *Note*: PTS Symptoms = Post-traumatic stress symptoms, *B* = unstandardized beta, *SE* = standard error, *β* = standardized beta, sr^2^ = semi-partial correlation. | | | | | | | | | |

| Table S9: *Regression Results with Perceived Social Support (Time 1) Predicting Avoidance- and Arousal-Based PTS Symptoms (Time 2).* | | | | | | | | | |
| --- | --- | --- | --- | --- | --- | --- | --- | --- | --- |
|  |  | *B* | *SE* | *β* | *p-value* | *95% CI* | | *sr^2^* | *R^2^* |
|  |  |  |  |  |  | Lower | Upper |  |  |
| Step 1 | *F*(2,81) = 31.10, *p* < .001 | | |  |  |  |  |  | 0.43 |
|  | Age | -0.01 | 0.04 | -0.02 | .752 | -0.10 | 0.08 | .00 |  |
|  | PTS Symptoms (Time 1) | 0.66 | 0.09 | 0.66 | <.001 | 0.50 | 0.83 | .42 |  |
| Step 2 | *F*(3,80) = 24.44, *p* < .001 | | |  |  |  |  |  | 0.48 |
|  | Age | -0.05 | 0.05 | -0.09 | .294 | -0.14 | 0.04 | .01 |  |
|  | PTS Symptoms (Time 1) | 0.62 | 0.08 | 0.61 | <.001 | 0.45 | 0.78 | .36 |  |
|  | Perceived Social Support (Time 1) | -1.53 | 0.59 | -0.22 | .011 | -2.71 | -0.36 | .05 |  |
| Step 3 | *F*(4,79) = 19.17, *p* < .001 | | |  |  |  |  |  | 0.49 |
|  | Age | -0.04 | 0.05 | -0.08 | .345 | -0.13 | 0.05 | .01 |  |
|  | PTS Symptoms (Time 1) | 0.61 | 0.08 | 0.60 | <.001 | 0.44 | 0.77 | .35 |  |
|  | Perceived Social Support (Time 1) | -1.60 | 0.59 | -0.23 | .008 | -2.77 | -0.43 | .05 |  |
|  | Cohort | 1.61 | 1.08 | 0.12 | .139 | -0.53 | 3.75 | .01 |  |
| Step 4 | *F*(5,78) = 18.10, *p* < .001 | | |  |  |  |  |  | 0.54 |
|  | Age | -0.03 | 0.04 | -0.06 | .478 | -0.12 | 0.06 | .00 |  |
|  | PTS Symptoms (Time 1) | 0.58 | 0.08 | 0.57 | <.001 | 0.42 | 0.74 | .30 |  |
|  | Perceived Social Support (Time 1) | -1.51 | 0.57 | -0.22 | .009 | -2.64 | -0.39 | .04 |  |
|  | Cohort | 1.66 | 1.04 | 0.13 | .113 | -0.40 | 3.72 | .01 |  |
|  | Cohort*Perceived Social Support | 2.97 | 1.08 | 0.22 | .008 | 0.81 | 5.13 | .04 |  |
| *Note*: PTS Symptoms for this model only include avoidance and arousal (Cluster C and E) symptoms. PTS Symptoms = Post-traumatic stress symptoms, *B* = unstandardized beta, *SE* = standard error, *β* = standardized beta, *sr^2^* = semi-partial correlation. | | | | | | | | | |

| Table S10: *Regression Results with Social Network Predicting Avoidance- and Arousal-Based PTS Symptoms (Time 2).* | | | | | | | | | |
| --- | --- | --- | --- | --- | --- | --- | --- | --- | --- |
|  |  | *B* | *SE* | *β* | *p-value* | *95% CI* | | *sr^2^* | *R^2^* |
|  |  |  |  |  |  | Lower | Upper |  |  |
| Step 1 | *F*(2,81) = 31.10, *p* < .001 | | |  |  |  |  |  | .43 |
|  | Age | -0.01 | 0.05 | -0.03 | .752 | -0.10 | 0.08 | .00 |  |
|  | PTS Symptoms (Time 1) | 0.66 | 0.09 | 0.66 | <.001 | 0.50 | 0.83 | .42 |  |
| Step 2 | *F*(3,80) = 21.16, *p* < .001 | | |  |  |  |  |  | .44 |
|  | Age | -0.02 | 0.05 | -0.04 | .653 | -0.11 | 0.07 | .00 |  |
|  | PTS Symptoms (Time 1) | 0.67 | 0.09 | 0.66 | <.001 | 0.50 | 0.84 | .42 |  |
|  | Social Network | -0.42 | 0.39 | -0.09 | .285 | -1.20 | 0.36 | .01 |  |
| Step 3 | *F*(4,79) = 16.27, *p* < .001 | | |  |  |  |  |  | .45 |
|  | Age | -0.02 | 0.05 | -0.03 | .748 | -0.11 | 0.08 | .00 |  |
|  | PTS Symptoms (Time 1) | 0.66 | 0.09 | 0.65 | <.001 | 0.49 | 0.83 | .42 |  |
|  | Social Network | -0.38 | 0.39 | -0.08 | .337 | -1.16 | 0.40 | .01 |  |
|  | Cohort | 1.29 | 1.12 | 0.10 | .252 | -0.94 | 3.52 | .01 |  |
| Step 4 | *F*(5,78) = 17.69, *p* < .001 | | |  |  |  |  |  | .53 |
|  | Age | 0.00 | 0.04 | 0.01 | .925 | -0.08 | 0.09 | .00 |  |
|  | PTS Symptoms (Time 1) | 0.69 | 0.08 | 0.68 | <.001 | 0.53 | 0.85 | .46 |  |
|  | Social Network | -0.30 | 0.37 | -0.07 | .409 | -1.03 | 0.42 | .01 |  |
|  | Cohort | 1.32 | 1.04 | 0.10 | .208 | -0.75 | 3.40 | .01 |  |
|  | Cohort*Social Network | 2.66 | 0.73 | 0.29 | <.001 | 1.21 | 4.11 | .08 |  |
| *Note*: PTS Symptoms for this model only include avoidance and arousal (Cluster C and E) symptoms. PTS Symptoms = post-traumatic stress symptoms, *B* = unstandardized beta, *SE* = standard error, *β* = standardized beta, *sr^2^* = semi-partial correlation. | | | | | | | | | |

| Table S11: *Regression Results with Social Constraints Predicting Avoidance- and Arousal-Based PTS Symptoms (Time 2).* | | | | | | | | | |
| --- | --- | --- | --- | --- | --- | --- | --- | --- | --- |
|  |  | *B* | *SE* | *β* | *p-value* | *95% CI* | | *sr^2^* | *R^2^* |
|  |  |  |  |  |  | Lower | Upper |  |  |
| Step 1 | *F*(2,81) = 31.10, *p* < .001 | | |  |  |  |  |  | .43 |
|  | Age | -0.01 | 0.05 | -0.03 | .752 | -0.10 | 0.08 | .00 |  |
|  | PTS Symptoms (Time 1) | 0.66 | 0.09 | 0.66 | <.001 | 0.50 | 0.83 | .42 |  |
| Step 2 | *F*(3,80) = 23.26, *p* < .001 | | |  |  |  |  |  | .47 |
|  | Age | -0.03 | 0.04 | -0.06 | .513 | -0.12 | 0.06 | .00 |  |
|  | PTS Symptoms (Time 1) | 0.59 | 0.09 | 0.59 | <.001 | 0.42 | 0.77 | .29 |  |
|  | Social Constraints | 1.94 | 0.90 | 0.19 | .033 | 0.16 | 3.73 | .03 |  |
| Step 3 | *F*(4,79) = 18.11, *p* < .001 | | |  |  |  |  |  | .48 |
|  | Age | -0.02 | 0.04 | -0.04 | .595 | -0.11 | 0.07 | .00 |  |
|  | PTS Symptoms (Time 1) | 0.58 | 0.09 | 0.58 | <.001 | 0.41 | 0.76 | .28 |  |
|  | Social Constraints | 2.00 | 0.89 | 0.20 | .028 | 0.22 | 3.77 | .03 |  |
|  | Cohort | 1.50 | 1.09 | 0.11 | .173 | -0.67 | 3.67 | .01 |  |
| Step 4 | *F*(5,78) = 15.45, *p* < .001 | | |  |  |  |  |  | .50 |
|  | Age | -0.02 | 0.04 | -0.03 | .712 | -0.10 | 0.07 | .00 |  |
|  | PTS Symptoms (Time 1) | 0.57 | 0.09 | 0.57 | <.001 | 0.40 | 0.75 | .27 |  |
|  | Social Constraints | 1.50 | 0.93 | 0.15 | .108 | -0.34 | 3.34 | .02 |  |
|  | Cohort | 1.51 | 1.08 | 0.11 | .164 | -0.63 | 3.65 | .01 |  |
|  | Cohort*Social Constraints | -3.00 | 1.74 | -0.15 | .088 | -6.46 | 0.45 | .02 |  |
| *Note*: PTS Symptoms for this model only include avoidance and arousal (Cluster C and E) symptoms. PTS Symptoms = post-traumatic stress symptoms, *B* = unstandardized beta, *SE* = standard error, *β* = standardized beta, *sr^2^* = semi-partial correlation. | | | | | | | | | |
